# Numerical Study and Optimisation of a Novel Single-Element Dual-Frequency Ultrasound Transducer

**DOI:** 10.3390/s18030703

**Published:** 2018-02-27

**Authors:** Changhe Sun, Senlin Jiang, Yufei Liu

**Affiliations:** 1 Key Laboratory of Optoelectronic Technology & Systems, Chongqing University, Ministry of Education, Chonqing 400044, China; chhesun@cqu.edu.cn (C.S.); jiangsenlin@cqu.edu.cn (S.J.); 2 Centre for Intelligent Sensing Technology, College of Optoelectronic Engineering, Chongqing University, Chongqing 400044, China; 3 Collaborative Innovation Center for Brain Science, Chongqing University, Chongqing 400044, China

**Keywords:** dual-frequency ultrasound transducer, single-element, piezoelectric-capacitive hybrid structure, equivalent circuit, finite element simulation

## Abstract

A dual-frequency ultrasound transducer (DFUT) is usually preferred for its numerous advantageous applications, especially in biomedical imaging and sensing. However, most of DFUTs are based on the combination of fundamental and harmonic operations, or integration of multiple different single-frequency ultrasound transducers, hindering perfect beam alignment and acoustic impedance matching. A novel single-element DFUT has been proposed in this paper. A small piezoelectric membrane is used as the high-frequency ultrasound transducer, which is stacked on a large non-piezoelectric elastic membrane with a groove used as the low-frequency capacitive ultrasound transducer. Such a capacitive-piezoelectric hybrid structure is theoretically analysed in details, based on the electrostatic attraction force and converse piezoelectric effect. Both the low and high resonance frequencies are independently derived, with a maximum deviation of less than 4% from the finite element simulations. Besides, a lumped-parameter equivalent circuit model of combining both the capacitive and piezoelectric ultrasound transducers was also described. Based on our dual-frequency structure design, a high-to-low frequency ratio of about 2 to more than 20 could be achieved, with easy and independent controllability of two frequencies, and the high-frequency operation shows at least an order-of-magnitude displacement sensitivity improvement compared with the conventional harmonic operations.

## 1. Introduction

Benefiting from deep penetration and high resolution, dual-frequency ultrasound transducers (DFUTs) have been studied in recent years for applications in medical therapy and imaging, such as non-destructive testing, transdermal drug release, photoacoustic imaging, and acoustic cavitation enhancement [1,2,3,4,5]. In order to achieve a dual-frequency ultrasound operation, so far there are four main approaches: (1) interleaving two different single-frequency elements in the horizontal plane of one chip [6,7,8,9], (2) stacking two different single-frequency elements in the vertical plane of one chip [10,11,12,13,14], (3) combining the thickness-bending (TB) and thickness-expansion (TE) modes in one transducer element [15], and (4) combining the fundamental and harmonic/superharmonic vibration modes in one transducer element [16,17,18]. In general, the first two DFUT configurations typically tend to be adopted because of their advantages in individual optimization for each frequency band and low coupling between the adjacent elements.

When two ultrasound transducer elements with different operating frequencies are arranged horizontally, to minimize deleterious grating lobes and ensure ideal co-registration, it is desirable that the low and high frequency transducer elements are spaced within a pitch, which is no more than half the high frequency acoustic wavelength (λ/2). Zemp et al. designed a dual-frequency capacitive micromachined ultrasound transducer (CMUT) 2D array with interlaced low-frequency and high-frequency elements [7]. Though they achieved favorable co-registered beams, the pitch between composite elements is just on a scale of λ for high-frequency (6.5 MHz) operation and the linear array design based on such composite elements would be challengeable for the perfect dual-frequency beam alignment. On the other hand, when two transducer elements with different operating frequencies are stacked vertically, they share the same footprint and have a competitive advantage of closely overlapping transmit/receive beams. Unfortunately, almost all present stacked DFUT designs are based on piezoelectric technology which is largely limited by the strict acoustic impedance matching, low electromechanical coupling factor, and weak compatibility with CMOS process. Additionally, the resonance frequencies of the stacked dual-layer piezoelectric transducers are easily shifted due to the aliasing echoes from the backside layer. A frequency selective isolation layer (FSIL) between two elements is preferably used to eliminate the crosstalk [10,11]. Manh et al. designed a dual-frequency capacitive-piezoelectric hybrid ultrasound transducer consisting of a low-frequency piezoelectric stack and a high-frequency CMUT served as an outer matching layer for the low-frequency band [13]. Through combining both the capacitive and piezoelectric ultrasound transducer technologies, it may be an innovative and effective approach to correcting the impedance mismatching, but it also inevitably leads to the reverberations from the back layer to the top CMUT.

A single-element DFUT with the hybrid vibration modes could overcome almost all inherent drawbacks of abovementioned interleaved or stacked DFUTs. Considering the combination of the TB and TE vibration modes for one piezoelectric plate, it is a promising method to design a single-element ultrasound transducer for dual-frequency operations. However, a principal difficulty in balancing the TB and TE resonance frequencies is that the thickness of the piezoelectric plate has a linearly proportional influence on the TB mode but an inversely proportional influence on the TE mode. As a result, this kind of piezoelectric device suffers from the conflicting requirements of thick piezoelectric plate used for TE mode, thin plate used for TB mode, and well piezoelectric process technologies, especially for medical ultrasound therapy and imaging applications. Hedegaard et al. showed a feasible design with λ-pitched piezoelectric micromachined ultrasound transducer (PMUT) arrays fabricated using a thick-film screen printing process, in which the total thickness of membranes is 70 μm, and a combination operation of 6.8 MHz TB mode and 23.7 MHz TE mode has been achieved successfully [15]. Nevertheless, the acoustic impedance matching for two different frequency bands is nontrivial and the further experiments for therapy-imaging applications have not been conducted yet. As an alternative, a single-element DFUT can also be achieved by adopting the fundamental and harmonic vibration modes. Liu et al. proposed a dual-frequency system to enhance acoustic cavitation by stimulating the fundamental and harmonic waves of a single piezoelectric transducer simultaneously [16]. Since the harmonic/super-harmonic imaging, based on the acoustic cavitation effect, heavily relies on the nonlinear transient responses of contrast agents and/or tissues, and has low signal-to-noise ratio due to various background noises, this technology is mainly limited by the low spatial resolution and poor characteristics of high harmonics. In addition, by patterning the top electrodes into several segments and activating fundamental and harmonic frequencies through the switchable control circuit, the dual/multi-frequency ultrasound operation can be also realized [17]. However, the higher frequencies can be only generated within a narrow frequency range and the electrically switching integrated circuit is relatively complicated.

What’s more, an accurate mathematical model for the DFUT is preferably required to explore the two operating frequencies and guide for the structure design. Although massive theoretical models have been reported for pure PMUTs or CMUTs, most of previous researches are focused on circular ultrasound transducers and to our knowledge none of the existing models contain both the piezoelectric and capacitive ultrasound operations [19,20,21,22,23]. Cour et al. modelled CMUTs with square anisotropic plates using the full anisotropic equation and obtained the deflection expression of square plates with less than 0.1% deviation from the simulations in the central deflection [24]. However, there is a lack of analysis focused on the resonance frequency which crucially depends on an accurate calculation of the deflection. 

Herein, a novel single-element DFUT without harmonic operations is proposed, in which a small piezoelectric membrane served as the high-frequency PMUT is stacked on a large elastic membrane with a groove served as the low-frequency CMUT. The architecture of the DFUT device is detailed in Section 2. The rest of this paper is organized as follows: in Section 3, the analytical models for the deflection and resonance frequencies are presented, and a lumped-parameter equivalent circuit model for the capacitive-piezoelectric hybrid structure is described. In Section 4, calculation and simulation results are given and discussed in detail. Finally, conclusions are provided in Section 5. The originalities and main contributions in our work can be summarized as follows:

- Through etching a small groove in the center of one elastic membrane, an additional mechanical regime is entered, providing a new high-frequency fundamental vibration mode instead of harmonic modes.
- Combining the capacitive and piezoelectric ultrasound transducers, a novel single-element DFUT without harmonic operations is designed, with easy and independent controllability of two frequencies.
- Mathematical models and equivalent circuits are described to predict two operating frequencies of this single-element DFUT and optimize the structure for particular applications.

## 2. Design of Capacitive-Piezoelectric Hybrid DFUT

### 2.1. Hybrid DFUT Device Concept

**Figure 1:**
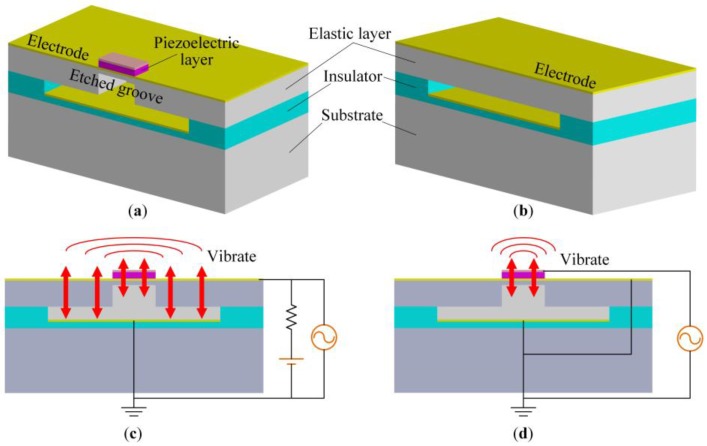
3D schematic structure (cross-sectional view) and operation modes, (**a**) the capacitive-piezoelectric hybrid DFUT; (**b**) one conventional CMUT for reference; (**c**) the low-frequency CMUT excitation form; (**d**) the high-frequency PMUT excitation form.

A 3D schematic structure (cross-sectional view) of a single DFUT element is shown in Figure 1a. The device mainly consists of one piezoelectric layer, one nonpiezoelectric elastic layer with an inner etched groove, a capacitive vacuum cavity, and three conductive metal layers: top electrode, middle electrode, and bottom electrode. In brief, this DFUT structure is a combination of one conventional CMUT and one typical PMUT stacked on the CMUT. One conventional CMUT is configured for reference as shown in Figure 1b. Through etching a square groove in the DFUT, the central part of the elastic layer is significantly softened, and a thin elastic membrane is left for supporting the piezoelectric layer. As the flexural rigidity of the outer elastic membrane is much larger than that of the central laminated layers, the outer elastic membrane roughly defines the square-shaped clamped boundaries for the central laminated layers. Thus, apart from the basic CMUT operation mode, an additional mechanical regime is entered: only the central piezoelectric laminated layers will vibrate when the DFUT is excited at the resonance frequency of the central laminated layers (named PMUT operation mode). The top and middle electrodes are deposited on the piezoelectric and elastic layers, respectively, and the bottom electrode is formed on the insulating layer. The vacuum cavity is sandwiched between the elastic layer and the bottom electrode, served as the variable capacitor.

### 2.2. Working Principle

A single DFUT element functions as a combination of one independent low-frequency CMUT and one independent high-frequency PMUT, both of which can be used as the transmitter and receiver. When operating in transmitting mode, by applying a low-frequency alternating-current (AC) voltage with the biased direct-current (DC) voltage between the middle and bottom electrodes, the whole elastic membranes will be driven to vibrate under the alternative electrostatic force and emit the acoustic waves into the surrounding medium, whereas, by applying a high-frequency AC voltage between the top and middle electrodes, only the central piezoelectric laminated membranes will deform and vibrate at the thickness-bending mode due to the converse piezoelectric effect, emitting acoustic waves into the surrounding medium. These two forms of electrical excitations in transmitting mode are shown in Figure 1c,d. On the contrary, by introducing a low-frequency or high-frequency incident ultrasound pressure into the single-element DFUT, the device will operate in receiving mode.

**Figure 2:**
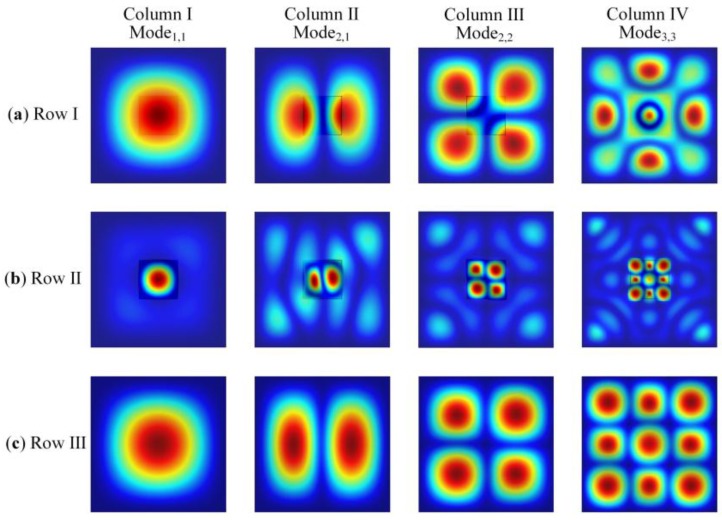
COMSOL Multiphysics modal simulations for the single-element DFUT, (**a**) at the CMUT operation mode; (**b**) at the PMUT operation mode; (**c**) for the referenced conventional CMUT.

In order to illustrate two different excitation forms of this single-element DFUT, finite element models are created by COMSOL Multiphysics v5.2a to simulate the modal shapes and compared with those of the conventional CMUT. Figure 2a shows the first and higher-order TB modes of the single-element DFUT in the CMUT excitation form and Figure 2b shows the similar TB modes of the DFUT in the PMUT excitation form, compared with the corresponding TB vibration modes of one conventional CMUT for reference as shown in Figure 2c. From the simulation results, it is clearly shown that three rows of TB vibration modes for the DFUT and conventional CMUT have the similar modal shapes. Therefore, the presented DFUT is capable of operating not only at the CMUT operation mode, but also at the PMUT operation mode without any other detrimental overtones disturbing. Aside from the fundamental vibration mode for the PMUT operation, the higher-order vibration modes could also be excited, however, it may not be suggested because they have lower acoustic coupling efficiency and are more likely to be affected by overtones and harmonic distortion. On the other hand, the PMUT operation frequencies of the single-element DFUT can be adjusted by changing the size of the etched groove. When the length of the etched groove gets increased, the resonance frequencies for the PMUT operation will be shifted down, meanwhile, the resonance frequencies for the CMUT operation mode will be shifted up slightly, which will be demonstrated in detail in Section 3 and Section 4. Therefore, the high-to-low frequency ratio for the presented DFUT can be realized within a certain range. To analyse two operation frequencies of the single-element DFUT, theoretical modelling, equivalent circuits and more detailed simulations should be performed prior to device fabrication.

## 3. Theoretical Analysis and Modelling

**Figure 3:**
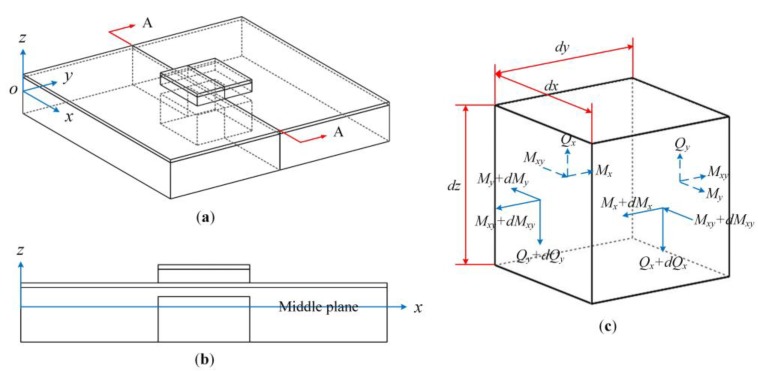
(**a**) The geometry of the single-element DFUT structure; (**b**) the cross-sectional view; (**c**) resultant forces and moments for one finite element.

Here the capacitive-piezoelectric hybrid DFUT is considered as a multilayered combination of a large nonpiezoelectric elastic plate with a small square groove inside and a small piezoelectric plate sandwiched between two metal electrodes, as shown in Figure 3. The structural parameters of this DFUT are defined in the glossary of symbols, as shown in Table 1. The outer boundaries of the large elastic layer are assumed to be fixed. The thickness-bending mode vibrations of the laminated plates can be induced when a sinusoidal input voltage is applied. Following the assumptions of Kirchhoff plate theory [25], the coordinate system is selected in a way that the axes x and y are in the middle plane of the laminated plates, whereas the axis z is normal to the middle plane. 

### 3.1. Motion Equations of the Laminated Plates

According to the Kirchhoff plate theory, the middle plane does not deform along its length and the displacement of any point along the normal line in the direction normal to plate is the same, thus the displacement *w*, *u*, and *v* of the laminated layers in *x*, *y*, and *z* directions can be expressed as [26,27]:

(1) $$\begin{array}{l} w(x,y,x)=w_{0}(x,y)\\ u(x,y,z)=u_{0}(x,y)-z\frac{\partial w(x,y)}{\partial x}\\ v(x,y,z)=v_{0}(x,y)-z\frac{\partial w(x,y)}{\partial y} \end{array}$$

where *w*_0_, *u*_0_, and *v*_0_ are the mid-plane displacements. 

Considering small deformations, the laminated strains can be written in terms of the mid-plane displacements as follows:

(2) $$\left(\begin{array}{c} \varepsilon_{x}\\ \varepsilon_{y}\\ \gamma_{xy} \end{array}\right)=\left(\begin{array}{c} \frac{\partial u}{\partial x}\\ \frac{\partial v}{\partial y}\\ \frac{\partial u}{\partial y}+\frac{\partial v}{\partial x} \end{array}\right)=\left(\begin{array}{c} \frac{\partial u_{0}}{\partial x}\\ \frac{\partial v_{0}}{\partial y}\\ \frac{\partial u_{0}}{\partial y}+\frac{\partial v_{0}}{\partial x} \end{array}\right)-z\left(\begin{array}{c} \frac{\partial^{2}w}{\partial x^{2}}\\ \frac{\partial^{2}w}{\partial x^{2}}\\ 2\frac{\partial^{2}w}{\partial x\partial y} \end{array}\right)=\varepsilon_{0}+z\kappa$$

where ***ε*_0_** and ***κ*** denote the mid-plane strain vector and plate curvature vector, respectively.

Let us consider an element cut out of the laminated plates by two pairs of planes parallel to the *xz* and *yz* planes, as shown in Figure 3c. Taking into consideration the small changes of the bending moments (*M_x_*, *M_y_*) and twisting moments (*M_xy_*), and vertical shearing forces (*Q_x_*, *Q_y_*) acting on the sides of the element when the coordinates *x* and *y* change by small quantities *dx* and *dy*, the following equations of equilibrium can be obtained:

(3) $$\left\{ \begin{array}{l} \frac{\partial \tau_{xz}}{\partial x}+\frac{\partial \tau_{yz}}{\partial y}+q-\rho \frac{\partial^{2}w}{\partial t^{2}}=0\\ z\frac{\partial \sigma_{x}}{\partial x}+z\frac{\partial \tau_{xy}}{\partial y}-\tau_{xz}=0\\ z\frac{\partial \tau_{xy}}{\partial x}+z\frac{\partial \sigma_{y}}{\partial y}-\tau_{yz}=0 \end{array}\right.$$

where *q* denotes the intensity of the uniform load distributed over the upper surfaces of plates.

After simplification, a single relationship among the three moment components can be found in the following form:

(4) $$\int z\left(\frac{\partial^{2}\sigma_{x}}{\partial x^{2}}+2\frac{\partial^{2}\tau_{xy}}{\partial x\partial y}+\frac{\partial^{2}\sigma_{y}}{\partial y^{2}}\right)dz-\int \rho \frac{\partial^{2}w}{\partial t^{2}}dz+q=0$$

A transversely isotropic material, which includes anisotropic piezoelectric materials like aluminum nitride (AlN), thermoelastic material silicon (Si) and silicon nitride (SiN_x_), is chosen as a trial here, which has only five independent elastic constants and three independent piezoelectric coefficients. Note that the interaction between normal stresses and shearing strains, shearing stresses and normal strains as well as shearing stresses and shearing strains can be ignored for the thin plates, the stress-strain relation and piezoelectric coefficients matrix can then be written as [28]:

(5) $$\left(\begin{array}{c} \varepsilon_{x}\\ \varepsilon_{y}\\ \gamma_{xy} \end{array}\right)=\left(\begin{array}{ccc} s_{11} & s_{12} & 0\\ s_{12} & s_{11} & 0\\ 0 & 0 & 2(s_{11}-s_{12}) \end{array}\right)\left(\begin{array}{c} \sigma_{x}\\ \sigma_{y}\\ \tau_{xy} \end{array}\right)$$

(6) $$\left(\begin{array}{c} D_{x}\\ D_{y}\\ D_{z} \end{array}\right)=\left(\begin{array}{cccccc} 0 & 0 & 0 & 0 & d_{15} & 0\\ 0 & 0 & 0 & d_{15} & 0 & 0\\ d_{31} & d_{31} & d_{33} & 0 & 0 & 0 \end{array}\right)\left(\begin{array}{c} \sigma_{x}\\ \sigma_{y}\\ \sigma_{z}\\ \tau_{yz}\\ \tau_{xz}\\ \tau_{xy} \end{array}\right)$$

According to mechanical and electrical boundary conditions that the transverse strain *ε_z_* is zero and *D_z_* is the only non-zero electric displacement component, the laminated plates will vibrate in the thickness-bending mode and the pertinent piezoelectric equations are given by:

(7) $$\left\{ \begin{array}{l} \varepsilon_{x}=s_{11}\sigma_{x}+s_{12}\sigma_{y}+d_{31}E_{z}\\ \varepsilon_{y}=s_{12}\sigma_{x}+s_{11}\sigma_{y}+d_{31}E_{z}\\ \gamma_{xy}=2(s_{11}-s_{12})\tau_{xy}\\ D_{z}=d_{31}\sigma_{x}+d_{31}\sigma_{y}+\varepsilon_{33}^{T}\varepsilon_{0}E_{z} \end{array}\right.$$

where $\varepsilon_{33}^{T}$ is the relative dielectric constant of the piezoelectric layer measured under constant stress and electric field *E_z_* = *V_tm/_t_p_* is applied across the top and bottom electrodes in the *z* direction, *V_tm_* is the applied voltage between the top and middle electrodes, *t_p_* is the thickness of the piezoelectric layer.

By expressing the stresses *σ_x_* and *σ_y_* in terms of the strains *ε_x_* and *ε_y_* using the piezoelectric constitutive equations, and then substituting them into the electric displacement expression, the stresses and electric displacement can be derived as:

(8) $$\left\{ \begin{array}{l} \sigma_{x}=\frac{s_{11}\varepsilon_{x}-s_{12}\varepsilon_{y}-\left(s_{11}-s_{12}\right)d_{31}E_{z}}{s_{11}^{2}-s_{12}^{2}}\\ \sigma_{y}=\frac{s_{11}\varepsilon_{x}-s_{12}\varepsilon_{y}-\left(s_{11}-s_{12}\right)d_{31}E_{z}}{s_{11}^{2}-s_{12}^{2}}\\ D_{z}=\frac{d_{31}}{s_{11}+s_{12}}\left(\varepsilon_{x}+\varepsilon_{y}\right)+{\bar{\varepsilon }}_{33}\varepsilon_{0}E_{z} \end{array}\right.$$

where ${\bar{\varepsilon }}_{33}=\varepsilon_{33}^{T}(1-k_{31}^{2})$ indicates the 2D cutoff dielectric constant, and $k_{31}^{2}=2d_{31}^{2}/(s_{11}+s_{12})\varepsilon_{33}^{T}\varepsilon_{0}$ is defined by the square of the electromechanical coupling factor of the laminated plates. 

Starting from Equations (2) and (8), the equilibrium Equation (4) can be written as a differential equation for the deflection *w* of the laminated plates:

(9) $$\int \frac{s_{11i}}{s_{11i}^{2}-s_{12i}^{2}}z^{2}\left(\frac{\partial^{4}w}{\partial x^{4}}+2\frac{\partial^{4}w}{\partial x^{2}\partial y^{2}}+\frac{\partial^{4}w}{\partial y^{4}}\right)dz+\int \rho_{i}\frac{\partial^{2}w}{\partial t^{2}}dz=q$$

where *i* denotes the *i*th layer from the bottom of the laminated layers (1st layer is the elastic layer, 2nd layer is the middle metal electrode, 3rd layer is the piezoelectric layer, 4th layer is the top metal electrode):

(10) $${w|}_{x=0}={w|}_{x=l}={w|}_{y=0}={w|}_{y=l}=0$$

(11) $${\frac{\partial w}{\partial x}|}_{x=0}={\frac{\partial w}{\partial x}|}_{x=l}={\frac{\partial w}{\partial x}|}_{y=0}={\frac{\partial w}{\partial x}|}_{y=l}=0$$

(12) $$\mathrm{Continuity}\;\mathrm{of}\;w,\;\frac{\partial w}{\partial x},\;\frac{\partial^{2}w}{\partial x^{2}},\;\mathrm{at}\;\mathrm{the}\;\mathrm{interface}\;\Gamma$$

where Γ is the interface between the large elastic plate and piezoelectric plate, defined by the outer boundaries of the piezoelectric plate.

In consideration of the discretization of the flexural rigidity for the laminated plates, the motion differential Equation (9) for the nonpiezoelectric region *R*_1_ and the piezoelectric region *R*_2_, can be redescribed by Equation (13a,b), respectively, as:

(13a) $$D_{1}\left(\frac{\partial^{4}w}{\partial x^{4}}+2\frac{\partial^{4}w}{\partial x^{2}\partial y^{2}}+\frac{\partial^{4}w}{\partial y^{4}}\right)+m_{1}\frac{\partial^{2}w}{\partial t^{2}}=q,\;(x,y)\in R_{1}$$

(13b) $$D_{2}\left(\frac{\partial^{4}w}{\partial x^{4}}+2\frac{\partial^{4}w}{\partial x^{2}\partial y^{2}}+\frac{\partial^{4}w}{\partial y^{4}}\right)+m_{2}\frac{\partial^{2}w}{\partial t^{2}}=q,\;(x,y)\in R_{2}$$

where *D*_1_ and *D*_2_ are the flexural rigidity of the laminated plates for the integration regions *R*_1_ and *R*_2_, respectively, $m_{1}$ and $m_{2}$ are the area plate density, defined as:

(14) $$\left(\begin{array}{c} D_{1}\\ D_{2} \end{array}\right)=\left(\begin{array}{c} \sum_{i=1}^{2}\frac{s_{11i}}{s_{11i}^{2}-s_{12i}^{2}}\int_{z_{i-1}-z_{m}}^{z_{i}-z_{m}}z^{2}dz\\ \sum_{j=1}^{4}\frac{s_{11j}}{s_{11j}^{2}-s_{12j}^{2}}\int_{z_{j-1}-z_{m}}^{z_{j}-z_{m}}z^{2}dz \end{array}\right),\;\left(\begin{array}{c} m_{1}\\ m_{2} \end{array}\right)=\left(\begin{array}{c} \sum_{i=1}^{2}\rho_{i}t_{i}\\ \sum_{j=1}^{4}\rho_{j}t_{j} \end{array}\right)$$

(15) $$z_{m}=\sum_{i=1}^{4}\frac{s_{11i}}{s_{11i}^{2}-s_{12i}^{2}}t_{i}(z_{i}-\frac{t_{i}}{2})/\sum_{i=1}^{4}\frac{s_{11i}}{s_{11i}^{2}-s_{12i}^{2}}t_{i}$$

### 3.2. Resonance Frequency

Due to the uncertainty of the deflection for the laminated plates, it is extremely difficult to directly determine the resonance frequencies from the motion differential Equation (13a,b). Therefore, the Galerkin method is preferably chosen to solve above equations by combining with the polynomial function to fit the deflection. Under the homogeneous voltage excitation with an electric potential magnitude *V* and an angular frequency *ω* = 2*πf*, the deflection can be assumed to take the form:

(16) $$w(x,y,t)=W(x,y)e^{i\omega t}$$

Based on previous trials [29,30,31], the residual stress could be reduced to zero by optimizing the fabrication process though the stress issue is hard to be eliminated in most cases. To simplify the analysis, the free vibration of the single-element DFUT is considered, thus the extra load term is neglected. Then the motion equations of the laminated plates become:

(17a) $$D_{1}\nabla^{2}\nabla^{2}W-m_{1}\omega^{2}W=0,\;(x,y)\in R_{1}$$

(17b) $$D_{2}\nabla^{2}\nabla^{2}W-m_{2}\omega^{2}W=0,\;(x,y)\in R_{2}$$

There are a set of determined resonance frequencies for various vibration mode shapes (*m*, *n*), defined by the number of modal lines *m* in the *x* direction and *n* in the *y* direction. For the clamped plates with the boundary conditions as Equations (10) and (11), the overall deflection *W*(*x*, *y*) is expressed by a set of constants *A_mn_* and mode-dependent polynomial functions *X_m_*(x) and *Y_n_*(*y*), which are set by means of separation of variables:

(18) $$W(x,y)=\sum_{m=1}^{\infty }\sum_{n=1}^{\infty }A_{mn}X_{m}(x)Y_{n}(y)$$

(19) $$X_{m}(x)=x^{2}\left[ml^{m+1}-(m+1)l^{m}x+x^{m+1}\right]$$

(20) $$Y_{n}(y)=y^{2}\left[nl^{n+1}-(n+1)l^{n}y+y^{n+1}\right]$$

With the polynomial approximation, the Galerkin method can be used to find the approximate solutions for the resonance frequencies of the DFUT [32]. Substituting Equations (18)–(20) into (17a,b), we select the polynomial functions *X_m_*(x) and *Y_n_*(*y*) as the weight function, and integration over the non-piezoelectric and piezoelectric regions for the motion equations is done as follows:

(21) $$\iint_{R_{1}+R_{2}}\left(D_{1}\nabla^{2}\nabla^{2}W-m_{1}\omega^{2}W\right)X_{m}Y_{n}dxdy+\iint_{R_{2}}\left[(D_{2}-D_{3})\nabla^{2}\nabla^{2}W-(m_{2}-m_{3})\omega^{2}W\right]X_{m}Y_{n}dxdy=0$$

where *D_3_* and $m_{3}$ are the flexural rigidity and area plate density of the etched groove when it is filled with the same elastic material, defined as:

(22) $$D_{3}=\frac{s_{111}}{s_{111}^{2}-s_{121}^{2}}\int_{-z_{m}}^{t_{1}-t_{s}-z_{m}}z^{2}dz,\;m_{3}=\rho_{1}t_{1}\;$$

Starting from Equation (21), the thickness-bending resonance frequency of the DFUT at CMUT operation can be derived as:

(23) $$f_{mn}=\frac{\omega_{mn}}{2\pi }=\frac{1}{2\pi }\sqrt{\frac{D_{1}L_{1}+\left(D_{2}-D_{3}\right)L_{2}}{m_{1}P_{11}Q_{11}+\left(m_{2}-m_{3}\right)P_{21}Q_{21}}}$$

where:

(24) $$\left(\begin{array}{c} L_{1}\\ L_{2} \end{array}\right)=\left(\begin{array}{c} P_{11}Q_{13}+2P_{12}Q_{12}+P_{13}Q_{11}\\ P_{21}Q_{23}+2P_{22}Q_{22}+P_{23}Q_{21} \end{array}\right)$$

(25) $$\left(\begin{array}{ccc} \begin{array}{l} P_{11}\\ P_{21} \end{array} & \begin{array}{l} P_{12}\\ P_{22} \end{array} & \begin{array}{l} P_{13}\\ P_{23} \end{array} \end{array}\right)=\left(\begin{array}{ccc} \int_{0}^{l}X_{m}\cdot X_{m}dy & \int_{0}^{l}\nabla^{2}X_{m}\cdot X_{m}dy & \int_{0}^{l}\nabla^{4}X_{m}\cdot X_{m}dy\\ \int_{\left(l-a\right)/2}^{\left(l+a\right)/2}X_{m}\cdot X_{m}dy & \int_{\left(l-a\right)/2}^{\left(l+a\right)/2}\nabla^{2}X_{m}\cdot X_{m}dy & \int_{\left(l-a\right)/2}^{\left(l+a\right)/2}\nabla^{4}X_{m}\cdot X_{m}dy \end{array}\right)$$

(26) $$\left(\begin{array}{ccc} \begin{array}{l} Q_{11}\\ Q_{21} \end{array} & \begin{array}{l} Q_{12}\\ Q_{22} \end{array} & \begin{array}{l} Q_{13}\\ Q_{23} \end{array} \end{array}\right)=\left(\begin{array}{ccc} \int_{0}^{l}Y_{m}\cdot Y_{m}dy & \int_{0}^{l}\nabla^{2}Y_{m}\cdot Y_{m}dy & \int_{0}^{l}\nabla^{4}Y_{m}\cdot Y_{m}dy\\ \int_{\left(l-a\right)/2}^{\left(l+a\right)/2}Y_{m}\cdot Y_{m}dy & \int_{\left(l-a\right)/2}^{\left(l+a\right)/2}\nabla^{2}Y_{m}\cdot Y_{m}dy & \int_{\left(l-a\right)/2}^{\left(l+a\right)/2}\nabla^{4}Y_{m}\cdot Y_{m}dy \end{array}\right)$$

Because the integration is performed over the entire region, the modal contours of the DFUT for CMUT operation can be plotted. At the fundamental thickness-bending vibration mode, the relative deflection of the laminated plates is given to be [24,33,34]:

(27) $$\frac{W(x,y)}{W_{0}}=\frac{256}{1+0.5\alpha }{\left(\frac{x}{l}\right)}^{2}{\left(1-\frac{x}{l}\right)}^{2}{\left(\frac{y}{l}\right)}^{2}{\left(1-\frac{y}{l}\right)}^{2}\times \left[1+\alpha \cdot \frac{x}{l}\left(1-\frac{x}{l}\right)+\alpha \cdot \frac{y}{l}\left(1-\frac{y}{l}\right)\right]$$

where *W*_0_ is the center deflection, *α* is the plate shape factor, related to the geometric parameters of the DFUT and the orientation of the anisotropic materials.

**Figure 4:**
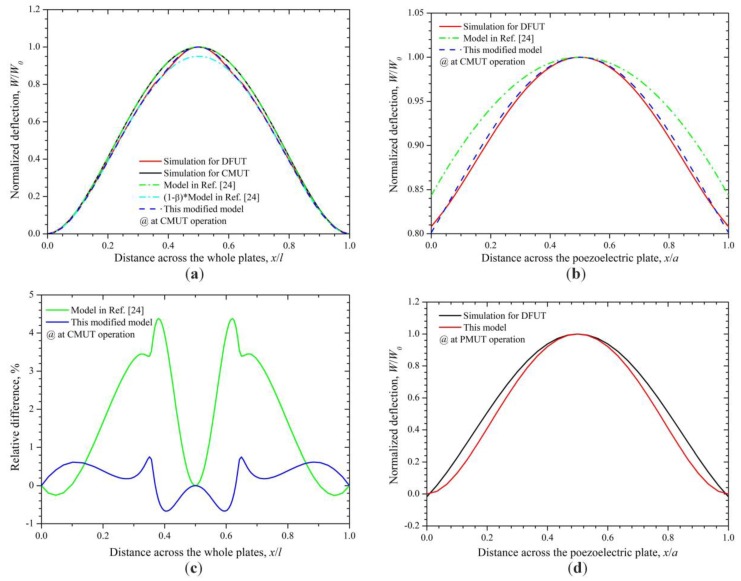
Normalized deflections (**a**) across the whole laminated plates and (**b**) across the piezoelectric plate for CMUT operation; (**c**) relative difference between theoretical calculations and simulations for CMUT operation; (**d**) normalized deflections across the piezoelectric plate for PMUT operation.

Let us consider a DFUT with the length ratio between the small and large plates of 2/7, the specific structural and material parameters of the DFUT are summarized in Table 2. The normalized deflection for the whole laminated plates in the *x* direction is shown in Figure 4a, obtained from the Equation (27) and COMSOL simulations, respectively. Compared with the normalized deflection of the conventional CMUT with the same size, the deviation from Equation (27) is less than 0.1%, whereas the deviation between Equation (27) and simulations for DFUT near the interface Γ reaches to around 4.4%, which may lead to a much larger error in the calculation of resonance frequencies. Therefore, the traditional deflection function for CMUT is unsuitable to the DFUT with an etched groove and a piezoelectric convex plate. To reduce such deviation, the deflection function may be modified as follows:

(28) $$f\left(x,y\right)=\left(1-\beta \right)\cdot x^{2}{\left(1-x\right)}^{2}y^{2}{\left(1-y\right)}^{2}+\frac{\varphi \left(x,y\right)\cdot \beta }{{\left(a/l\right)}^{8}}{\left(x-\frac{l-a}{2l}\right)}^{2}{\left(\frac{l+a}{2l}-x\right)}^{2}{\left(y-\frac{l-a}{2l}\right)}^{2}{\left(\frac{l+a}{2l}-y\right)}^{2}$$

where *f*(*x*, *y*) is the normalized deflection shape function, variables *x* and *y* substitute for *x*/*l* and *y*/*l* in Equation (27), *β* is the plate parameter of DFUT related to the geometric parameters and material properties of the device, *φ*(*x*, *y*) is the selecting function and defined by:

(29) $$\varphi \left(x,y\right)=\left\{ \begin{array}{l} 0,\;(x,y)\in R_{1}\\ 1,\;(x,y)\in R_{2} \end{array}\right.$$

Based on the modified deflection function, the analytical results are in excellent agreement with the COMSOL simulations (shown in Figure 4a), with a small difference from simulations less than 0.8% shown in Figure 4c. A zoom-in view on the piezoelectric region is shown in Figure 4b. In the further derivation, the fundamental thickness-bending resonance frequency of the DFUT at CMUT operation can be derived by substituting the modified deflection Equation (28) into Equation (21), as:

(30) $$f_{C1}=\frac{1}{2\pi l^{2}}\sqrt{\frac{2D_{1}(P_{C11}P_{C13}+P_{C12}^{2})+2(D_{2}-D_{3})(P_{C21}P_{C23}+P_{C22}^{2})}{m_{1}P_{C11}^{2}+(m_{2}-m_{3})P_{C21}^{2}}}$$

where:

(31) $$\left(\begin{array}{ccc} P_{C11} & P_{C12} & P_{C13}\\ P_{C21} & P_{C22} & P_{C23} \end{array}\right)={\left(\begin{array}{cc} \int_{0}^{1}X{\left(x\right)}^{2}dx & \int_{\left(l-a\right)/2l}^{\left(l+a\right)/2l}X{\left(x\right)}^{2}dx\\ \int_{0}^{1}\frac{\partial^{2}X(x)}{\partial x^{2}}\cdot X(x)dx & \int_{\left(l-a\right)/2l}^{\left(l+a\right)/2l}\frac{\partial^{2}X(x)}{\partial x^{2}}\cdot X(x)dx\\ \int_{0}^{1}\frac{\partial^{4}X(x)}{\partial x^{4}}\cdot X(x)dx & \int_{\left(l-a\right)/2l}^{\left(l+a\right)/2l}\frac{\partial^{4}X(x)}{\partial x^{4}}\cdot X(x)dx \end{array}\right)}^{T}$$

On the other hand, the resonance frequencies at PMUT operation can be obtained by assuming that boundaries of the central piezoelectric laminated plates are built-in as they are designed to have a much smaller mechanical flexural rigidity than that of the outer elastic plate. Therefore, only the piezoelectric laminated layers vibrate when excited by a specific high-frequency voltage source. The normalized deflection for the piezoelectric laminated plates in the *x* direction is shown in Figure 4d, obtained from Equation (27) and COMSOL simulations, respectively. Through applying the same calculation method to the PMUT operation, the fundamental thickness-bending resonance frequency then can be obtained in the form similar to that at CMUT operation:

(32) $$f_{P1}=\frac{12}{\pi a^{2}}\sqrt{\frac{D_{p}}{m_{1}}}$$

where *D_p_* is the flexural rigidity of the central piezoelectric laminated plates, *z_p_* is the distance from the neutral plane to the reference plane of the piezoelectric laminated plates, defined as:

(33) $$D_{p}=\sum_{i=1}^{4}\frac{s_{11i}}{s_{11i}^{2}-s_{12i}^{2}}\int_{-z_{p}}^{z_{i}-z_{p}}z^{2}dz$$

(34) $$z_{p}=\sum_{i=1}^{4}\frac{s_{11i}}{s_{11i}^{2}-s_{12i}^{2}}t_{i}(z_{i}-\frac{t_{i}}{2})/\sum_{i}^{4}\frac{s_{11i}}{s_{11i}^{2}-s_{12i}^{2}}t_{i}$$

### 3.3. Static Deflection

Starting from Equation (8), the piezoelectric interaction with the plates can be described with the in-plane strain variables *ε_x_* and *ε_y_*, which are the functions of the deflection. The axisymmetric deflection *W*(*x, y*) is the product of the static deflection *W*_0_ times the modified deflection shape function *f*(*x*, *y*). Thus, the electric displacement *D_z_* and the coupling energy of the electric Gibbs energy are given by:

(35) $$D_{z}=-\frac{d_{31p}}{s_{11p}+s_{12p}}z\left(\frac{\partial^{2}W}{\partial x^{2}}+\frac{\partial^{2}W}{\partial y^{2}}\right)+{\bar{\varepsilon }}_{33}\varepsilon_{0}\frac{V_{tm}}{t_{p}}$$

(36) $$U_{cp}=\underset{V_{2}}{∭}\frac{d_{31p}}{s_{11p}+s_{12p}}z\left(\frac{\partial^{2}W}{\partial x^{2}}+\frac{\partial^{2}W}{\partial y^{2}}\right)\frac{V_{tm}}{t_{p}}dxdydz=\frac{256W_{0}}{l^{2}}\cdot \frac{d_{31p}}{s_{11p}+s_{12p}}z_{cp}V_{tm}I_{cp}$$

where *z_cp_* is the distance from the middle plane of the piezoelectric layer to the neutral plane, *d*_31p_, *s*_11p_, *s*_12p_ and *t_p_* are the piezoelectric coefficient, compliance coefficients and thickness of the piezoelectric plate, *t_e_* is the thickness of metal conductive electrodes, *V*_2_ denotes the volume of the piezoelectric laminated plates, *I_cp_* is the coefficient, defined by:

(37) $$z_{cp}=t_{s}+t_{e}+\frac{t_{p}}{2}-z_{m}$$

(38) $$I_{cp}=\int_{\left(l-a\right)/2l}^{\left(l+a\right)/2l}\int_{\left(l-a\right)/2l}^{\left(l+a\right)/2l}\left(\frac{\partial^{2}f}{\partial x^{2}}+\frac{\partial^{2}f}{\partial y^{2}}\right)dxdy$$

Let us take the case that the laminated plates are bent by uniformly distributed bending moments *zσ_x_* and *zσ_y_* so that the *xz* and *yz* planes are the principal planes of the deflection surface of the plates, the strain energy stored in an element is obtained by calculating the work done by these moments on the element in the following form [35]:

(39) $$\begin{array}{ll} dU_{me} & =-\frac{1}{2}\left(z\sigma_{x}\frac{\partial^{2}W}{\partial x^{2}}+z\sigma_{y}\frac{\partial^{2}W}{\partial y^{2}}\right)dxdydz\\ & =\frac{s_{11}}{2\left(s_{11}^{2}-s_{12}^{2}\right)}z^{2}\left[{\left(\frac{\partial^{2}W}{\partial x^{2}}\right)}^{2}-2\frac{s_{12}}{s_{11}}\frac{\partial^{2}W}{\partial x^{2}}\frac{\partial^{2}W}{\partial y^{2}}+{\left(\frac{\partial^{2}W}{\partial y^{2}}\right)}^{2}\right]dxdydz \end{array}$$

By integrating Equation (39) over the entire volume of the plates, the total strain energy of the plates is:

(40) $$U_{me}=\frac{1}{2}\frac{{\left(256W_{0}\right)}^{2}}{l^{2}}\left(D_{1}\cdot I_{me1}+D_{2}\cdot I_{me2}\right)$$

where:

(41) $$\begin{array}{ll} I_{me1} & =\int_{0}^{1}\int_{0}^{1}\left[{\left(\frac{\partial^{2}f}{\partial x^{2}}\right)}^{2}-2\frac{s_{121}}{s_{111}}\frac{\partial^{2}f}{\partial x^{2}}\frac{\partial^{2}f}{\partial y^{2}}+{\left(\frac{\partial^{2}f}{\partial y^{2}}\right)}^{2}\right]dxdy\\ I_{me2} & =\int_{\left(l-a\right)/2l}^{\left(l+a\right)/2l}\int_{\left(l-a\right)/2l}^{\left(l+a\right)/2l}\left[{\left(\frac{\partial^{2}f}{\partial x^{2}}\right)}^{2}-2\frac{s_{12p}}{s_{11p}}\frac{\partial^{2}f}{\partial x^{2}}\frac{\partial^{2}f}{\partial y^{2}}+{\left(\frac{\partial^{2}f}{\partial y^{2}}\right)}^{2}\right]dxdy \end{array}$$

The electrical energy produced when operating at the PMUT operation and CMUT operation, respectively, is given by:

(42) $$U_{ep}=\frac{1}{2}C_{ep}V_{tm}^{2}=\frac{1}{2}\frac{{\bar{\varepsilon }}_{33p}\varepsilon_{0}a^{2}}{t_{p}}V_{tm}^{2}$$

(43) $$U_{ec}=\frac{1}{2}C_{ec}V_{mb}^{2}$$

where *ε*_0_ is the permittivity of the vacuum cavity, *C_ep_* and *C_ec_* are effective capacitance of the DFUT at PMUT and CMUT operation, respectively, *V_tm_* is the applied voltage across the top and middle electrodes, and *V_mb_* is the applied voltage across the middle and bottom electrodes.

Therefore, the complete electric Gibbs energy of the DFUT can be written as [30,36]:

(44) $$\begin{array}{ll} G & =U_{cp}+U_{me}+\left(U_{ep}-U_{ec}\right)\\ & =\frac{256W_{0}}{l^{2}}\cdot \frac{d_{31p}}{s_{11p}+s_{12p}}z_{cp}V_{tm}\cdot I_{cp}+\frac{1}{2}\frac{{\bar{\varepsilon }}_{33p}\varepsilon_{0}a^{2}}{t_{p}}V_{tm}^{2}+\frac{1}{2}\frac{{\left(256W_{0}\right)}^{2}}{l^{2}}\left(D_{1}\cdot I_{me1}+D_{2}\cdot I_{me2}\right)-\frac{1}{2}C_{ec}V_{mb}^{2} \end{array}$$

Then the equivalent spring constant can be obtained by using the Gibbs energy function (44), as:

(45) $$K_{eq}=\frac{\partial^{2}G}{\partial W_{0}^{2}}=K_{0}-\frac{1}{2}V_{mb}^{2}\frac{\partial^{2}C_{ec}}{\partial W_{0}^{2}}$$

where:

(46) $$K_{0}=\frac{256^{2}\left(D_{1}\cdot I_{me1}+D_{2}\cdot I_{me2}\right)}{l^{2}}$$

From Equation (45), a drop in the spring constant and resonance frequency of the DFUT device will be observed as *V_mb_* is increased gradually, which is highly in accordance with the spring softening phenomenon reported in [37,38,39]. Besides, it also suggests that there is a point at which the effective spring constant is zero as *V_mb_* is increased and the laminated plates collapses toward the bottom electrode. The collapse point occurs when the pull-in voltage reaches [24]:

(47) $$V_{PI}=\sqrt{\frac{2K_{0}}{\partial^{2}C_{ec}/\partial W_{0}^{2}}}$$

Furthermore, we assume that the electric excitation for the PMUT operation is neglected, i.e., $V_{tm}=0\;V$, by minimizing the Gibbs energy function (44), i.e., $\partial G/\partial W_{0}=0$, the center deflection *W*_0_ of the laminated plates reaches the maximum value and can be yielded as:

(48) $$W_{0}=\frac{V_{mb}^{2}}{2K_{0}}\cdot \frac{\partial C_{ec}}{\partial W_{0}}$$

The electrical capacitance of the DFUT at CMUT operation, as a function of the plate deflection, can be found:

(49) $$C_{ec}=\frac{C_{ec1}}{l^{2}-a^{2}}\iint_{R_{1}}\frac{1}{1-W\left(x,y\right)/\left(t_{g}+t_{1}/\varepsilon_{1}\right)}dxdy+\frac{C_{ec2}}{a^{2}}\iint_{R_{2}}\frac{1}{1-W\left(x,y\right)/\left(t_{g}+t_{1}-t_{s}+t_{s}/\varepsilon_{1}\right)}dxdy$$

where *C_ec_*_1_ and *C_ec_*_2_ are the capacitance at zero deflection, *t_g_* is the height of the capacitive vacuum cavity, *t_ge_*_1_ and *t_ge_*_2_ are the effective gap height of the capacitors *C_ec_*_1_ and *C_ec_*_2_, respectively, defined by:

(50) $$C_{ec1}=\frac{\varepsilon_{0}\left(l^{2}-a^{2}\right)}{t_{g}+t_{1}/\varepsilon_{1}}=\frac{\varepsilon_{0}\left(l^{2}-a^{2}\right)}{t_{ge1}},\;C_{ec2}=\frac{\varepsilon_{0}a^{2}}{t_{g}+t_{1}-t_{s}+t_{s}/\varepsilon_{1}}=\frac{\varepsilon_{0}a^{2}}{t_{ge2}}$$

Considering the case that the maximum deflection is far smaller than the effective distance between two metal electrodes of the capacitor, i.e., *W*_0_ << *t_ge_*_1_< *t_ge_*_2_, the total capacitance of the DFUT can then be derived, based on the second-order Taylor series expansion approximation:

(51) $$C_{ec}\approx \frac{C_{ec1}}{1-{\left(a/l\right)}^{2}}[1-\frac{a^{2}}{l^{2}}+\frac{W_{0}\left(g_{11}-g_{21}\right)}{t_{ge1}}+\frac{W_{0}^{2}\left(g_{12}-g_{22}\right)}{t_{ge1}^{2}}]+\frac{C_{ec2}}{{\left(a/l\right)}^{2}}\left(\frac{a^{2}}{l^{2}}+\frac{W_{0}g_{21}}{t_{ge2}}+\frac{W_{0}^{2}g_{22}}{t_{ge2}^{2}}\right)$$

where *g*_11_, *g*_12_, *g*_21_, and *g*_22_ are defined by:

(52) $$\left(\begin{array}{cc} g_{11} & g_{12}\\ g_{21} & g_{22} \end{array}\right)=\left(\begin{array}{cc} \int_{0}^{1}\int_{0}^{1}256f\left(x,y\right)dxdy & \int_{0}^{1}\int_{0}^{1}{\left[256f\left(x,y\right)\right]}^{2}dxdy\\ \int_{\left(l-a\right)/2l}^{\left(l+a\right)/2l}\int_{\left(l-a\right)/2l}^{\left(l+a\right)/2l}256f\left(x,y\right)dxdy & \int_{\left(l-a\right)/2l}^{\left(l+a\right)/2l}\int_{\left(l-a\right)/2l}^{\left(l+a\right)/2l}{\left[256f\left(x,y\right)\right]}^{2}dxdy \end{array}\right)$$

Then the center static deflection of the laminated plates can be represented by substituting Equation (51) into Equation (48):

(53) $$W_{0}=\frac{V_{mb}^{2}}{2K_{eq}}\cdot l^{2}\varepsilon_{0}\left(\frac{g_{11}-g_{21}}{t_{ge1}^{2}}+\frac{g_{21}}{t_{ge2}^{2}}\right)$$

where:

(54) $$K_{eq}=K_{0}-l^{2}\varepsilon_{0}\left(\frac{g_{12}-g_{22}}{t_{ge1}^{3}}+\frac{g_{22}}{t_{ge2}^{3}}\right)V_{mb}^{2}$$

### 3.4. Electrical Equivalent Circuits

Starting from Equation (54), the mechanical compliance, equivalent inductance and transformer ratio of the DFUT at CMUT operation can be expressed by:

(55) $$C_{c}=\frac{1}{K_{eq}},\;L_{c}=\frac{1}{{\left(2\pi f_{C1}\right)}^{2}C_{c}}$$

(56) $$n_{c}=\frac{\partial F}{\partial V_{mb}}=-V_{mb}\frac{\partial C_{c}}{\partial W_{0}}$$

(57) $$n_{p}=\frac{\partial F}{\partial V_{tm}}=\frac{d_{31p}z_{cp}}{s_{11p}+s_{12p}}I_{cp}$$

**Figure 5:**
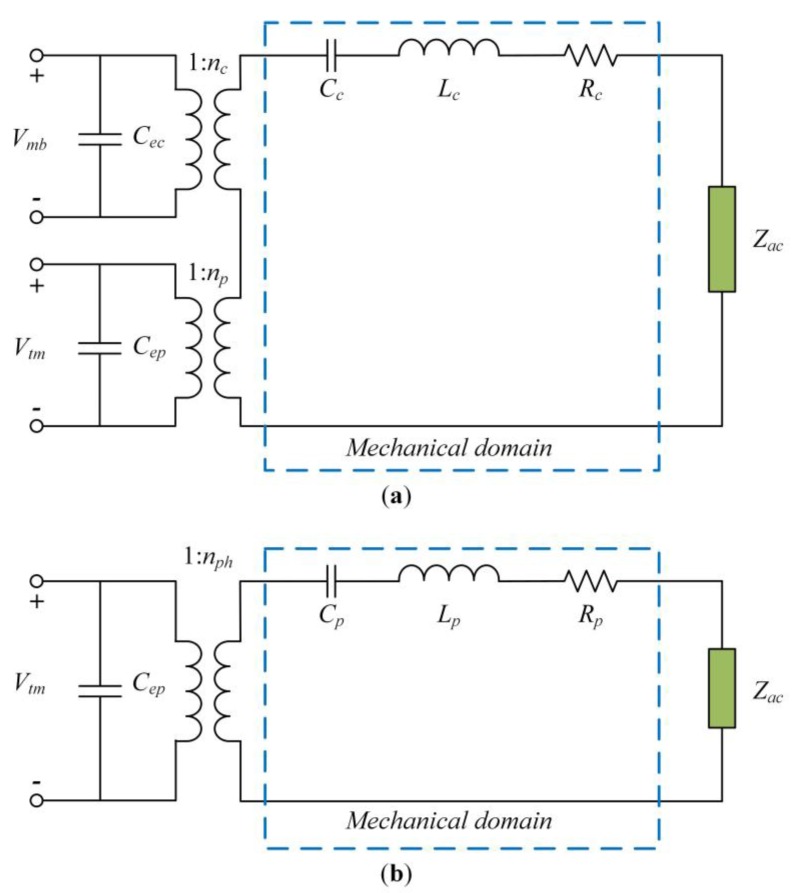
Electrical equivalent circuits of the DFUT, (**a**) at the CMUT operation; (**b**) at the PMUT operation.

Therefore, the frequency response of the DFUT at CMUT operation can be modelled when the mechanical resistance *R_c_* of the whole plates is given, and the electrical equivalent circuit model is described in Figure 5a.

However, when the DFUT operates as one PMUT, the above model will be inapplicable because only the central piezoelectric laminated plates vibrate and generate the acoustic waves when excited over some high-frequency band. Applying the same calculation method, the effective mechanical stiffness at PMUT operation can be derived as:

(58) $$K_{eq.p}=\frac{\partial^{2}G}{\partial W_{0}^{2}}=\frac{256^{2}D_{p}}{a^{2}}I_{me1}$$

Similarly, when the mechanical resistance *R_p_* of the central piezoelectric laminated plates is given, the mechanical compliance, equivalent inductance and transformer ratio of the DFUT at PMUT operation can be obtained, and the electrical equivalent circuit model for PMUT operation is described in Figure 5b, where *Z_ac_* is the acoustic impedance:

(59) $$C_{p}=\frac{1}{K_{eq.p}},\;L_{p}=\frac{1}{{\left(2\pi f_{P1}\right)}^{2}C_{p}}$$

(60) $$n_{ph}=\frac{\partial F}{\partial V_{tm}}=\frac{d_{31p}z_{cp}}{s_{11p}+s_{12p}}I_{cph}$$

where:

(61) $$I_{cph}=\int_{0}^{1}\int_{0}^{1}\left(\frac{\partial^{2}f}{\partial x^{2}}+\frac{\partial^{2}f}{\partial y^{2}}\right)dxdy$$

## 4. Results and Discussion

The analytical calculations and simulations for the single-element DFUT with 70 μm in length are employed as a specific example, in which the theoretical analysis is coded in MATLAB R2015b and the simulation is performed in COMSOL Multiphysics v5.2a. The structural dimensions and material properties of the DFUT can be found in Table 2. The height of the capacitive vacuum cavity is 500 nm.

**Figure 6:**
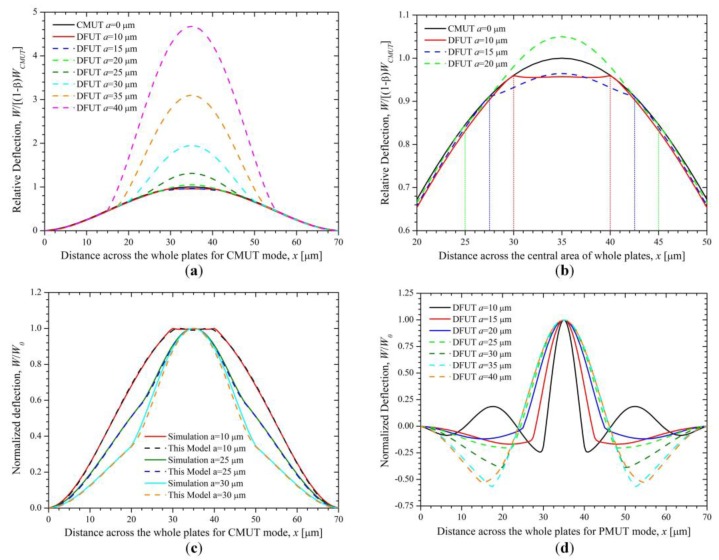
Relative deflection for CMUT mode, (**a**) across the whole plates; (**b**) a zoom-in view across the central region of the whole plates; (**c**) normalized deflection across the whole plates for CMUT mode; (**d**) normalized deflection across the whole plates for PMUT mode.

By changing the length of the etched square groove, the deflection along the length direction of the large elastic plate can be seen in Figure 6a. Because the central piezoelectric laminated plates are much thinner than the outer elastic plate, they are more likely to produce a large deformation with increasing the length of the groove. However, when the groove is smaller than 10 μm, the central laminated plates will be lifted up by the outer heavy plates and then be compressed downward by the induced stress. Actually, this stress is not high enough to bring a remarkable displacement so that the central laminated plates are a bit flat as shown in Figure 6b, compared with the outer elastic plate. On the other hand, the central laminated plates will be much softened and deflected when the length of the square groove is increased and larger than 15 μm, thus they may produce a larger displacement than the outer elastic plate. The normalized deflection of the DFUT at CMUT operation was mathematically modelled and simulated as shown in Figure 6c, where the plate parameter β used in our proposed model is summarized in Table 3. It is noticed that there is a deviation of no greater than 3.9% between the theoretical model and simulation, and in fact this deviation will be significantly reduced (≤1%) when the length of the groove is smaller than 30 μm. The normalized deflection for PMUT operation is also analyzed when the length of the groove is changed from 10 to 40 μm, as shown in Figure 6d. It can be seen that the ripples in the outer elastic plate become quite remarkable and their amplitude reaches more than one half of the wave displacement when the length of the groove is larger than 30 μm. This can be explained by comparative mechanical stiffness of the central piezoelectric laminated plates and outer elastic plate, and resultant comparative operation frequencies at CMUT and PMUT operations. Therefore, it is suggested the length of the designed groove should not be larger than one half of the length of the DFUT in order to achieve a desirable dual-frequency operation.

**Figure 7:**
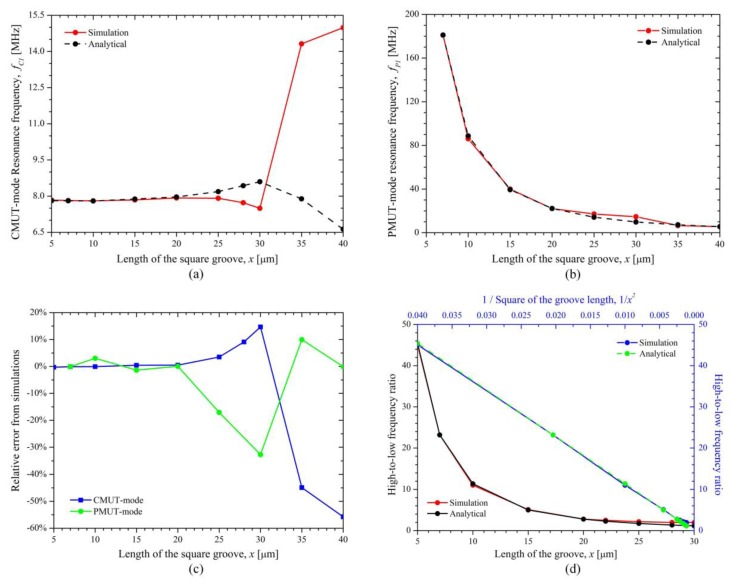
Fundamental resonance frequencies, (**a**) at CMUT operation; (**b**) at PMUT operation; (**c**) relative errors form simulations; (**d**) high-to-low frequency ratio of the DFUT when the length of the groove changes from 5 to 30 μm.

The fundamental thickness-bending resonance frequencies of the DFUT for CMUT and PMUT operations were analytically calculated and compared with their corresponding simulations. With various sizes of the square groove, the resonance frequencies for CMUT and PMUT operation are shown in Figure 7a,b, respectively. It is noticed that the frequencies at CMUT operation increases slightly when the length of the groove changes from 5 to 20 μm, and reaches to the peak value for the groove with 20 μm in length. On the contrary, the frequencies at PMUT operation drops quickly when the length of the groove is smaller than 20 μm, and then falls gradually to about 15 MHz which is very close to the second-order frequency of the CMUT operation when the length of the groove varies from 20 to 30 μm. Besides, when the size of the groove is larger than 30 μm, a remarkable opposite jump can be observed for CMUT and PMUT operations, thereby resulting in an exchange of high- and low-frequency modes. All these may be caused by vibration mode interaction between CMUT and PMUT operations, in consideration that the fundamental frequency at PMUT operation is quite close to the fundamental or second-order frequency at CMUT operation. Moreover, the results from Figure 7a,b also demonstrate the designed single-element DFUT is capable of generating both low- and high-frequency acoustic waves, and the theoretical calculations for dual frequencies are highly in accordance with the simulations when the length of the etched groove ranges from 5 to 25 μm. The deviation from the simulations is less than 4% (Figure 7c) and the errors are mainly due to the misshapen deflections and softened boundaries at PMUT operation. Thus, dual frequencies of this DFUT can be achieved predictably and accurately for the groove with a length of no larger than 25 μm. To further illustrate the dual-frequency property of the single-element DFUT, the high-to-low frequency ratio (HLFR) is defined as the ratio of the fundamental resonance frequencies at PMUT and CMUT operations, expressed by Equation (62):

(62) $$HLFR=\frac{f_{High}}{f_{Low}}=\frac{f_{P1}}{f_{C1}}$$

**Figure 8:**
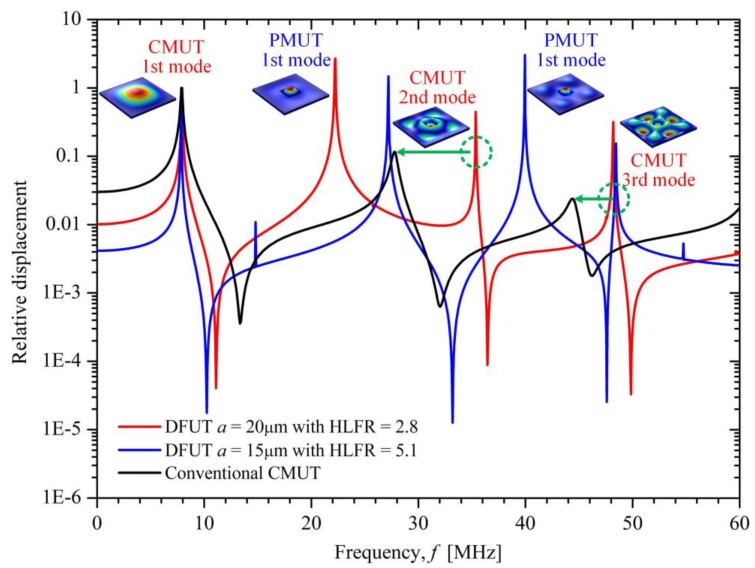
Simulated frequency response for the single-element DFUT and one conventional CMUT.

It can be seen from Figure 7d that the HLFR is approximately inversely proportional to the square of the length of the groove and can be achieved in a wide range from around 2 to over 20. For example, the HLFR is 1.98 for the etched groove with length of 28 μm which equals to 0.4 *l* (*l* is the length of DFUT), while it reaches to 23.18 for the groove with length of 7 μm which equals to 0.1*l*. Here the etched groove with a length of 20 μm is considered, so the HLFR of the DFUT is about 2.80. The simulated frequency responses for this novel DFUT and one conventional CMUT with the same fundamental frequency as a comparison are plotted in Figure 8. Simulations for the 70 μm DFUT perfectly show a low-frequency CMUT mode of 7.92 MHz and a high-frequency PMUT mode of 22.15 MHz, as aforementioned analytical model predicts. What’s more, the displacement amplitude of the high-frequency PMUT mode is in the same order of magnitude of the low-frequency CMUT mode, suggesting such dual-frequency operation has a unique advantage of at least one order of magnitude displacement sensitivity improvement when compared with the conventional harmonic/super-harmonic operations. In contrast, the high-frequency PMUT mode cannot be observed in the conventional CMUT except for its overtones. It should be noted that the second-order and third-order resonance frequencies of the CMUT operation are shifted upward due to the vibration mode interaction between CMUT and PMUT operations and modulated flexural rigidity when compared with the corresponding frequencies of the conventional CMUT. 

**Figure 9:**
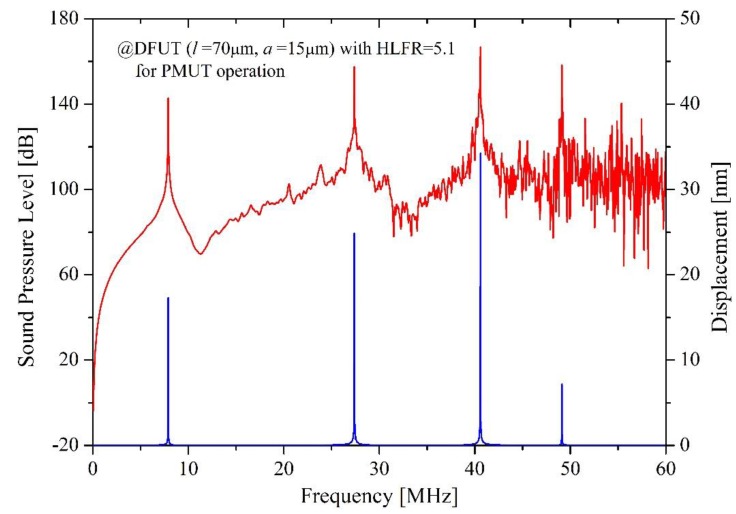
Ultrasound pressure generated near the top surface of the DFUT under 1V_pp_ electrical excitation.

In order to further demonstrate this innovative dual-frequency ultrasound operation, another DFUT with a 15 μm-length groove was also simulated to make a comparison, showing a higher HLFR of about 5.1 and good accordance with the above results. Besides, the sound pressure characteristic is also analyzed for this kind of DFUT. When the transducer is under 1V peak-to-peak voltage excitation, the sound pressure level (SPL) is above 160 dB at the 1st PMUT mode, and above 140 dB at the 1st CMUT mode, as shown in Figure 9.

**Figure 10:**
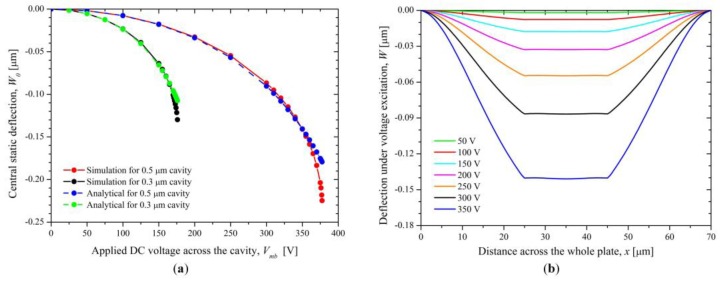
(**a**) Center static deflection and (**b**) deflection across the whole plates at CMUT operation under the DC voltage excitation.

Compared with the displacement analysis, the same frequency response can be observed. It is worth mentioning that the 2nd CMUT mode is also dominant, showing the SPL of about 160 dB and the displacement sensitivity of 25 nm/V_pp_. The reason why the higher-order harmonic signals are comparable to the fundamental signals is that the the etched groove and stacked piezoelectric membrane changes the mechanical characteristic of the whole membrane and results in the asymmetry of the flexural rigidity on the whole plane. Therefore, the dual-frequency ultrasound transducer proposed is also promising to operate over multiple frequency bands in consideration of enhanced second and third vibration modes When the DFUT with the length in 20 μm works as the CMUT, the static voltage characteristic is analyzed with the simulation. For the DFUT with a vacuum cavity height of 500 nm, the pull-in voltage is about 380 V, while it is about 180 V for the DFUT with a 300 nm vacuum cavity, as shown in Figure 10a. Under a small DC voltage *V_DC_* (<50% *V_PI_*) excitation, the center static deflection of the DFUT is plotted in Figure 10b. Besides, theoretical calculations are conducted for comparison with COMSOL simulations, showing a good agreement with the simulation results. However, the deviation from simulations becomes significant when the applied bias voltage is increased to larger than 90%*V_PI_*, this mainly resulted from the second-order Taylor series expansion approximation for the calculation of the central static deflection. 

## 5. Conclusions

In summary, a novel single-element DFUT was designed. Through etching a small groove in the center of an elastic membrane, the flexural rigidity of the membrane could be discretized. The outer part without covering the groove has a much larger flexural rigidity than that of the central part, thereby roughly defining the clamped boundaries for the central part and entering an additional mechanical regime: only the central part rather than the whole membrane will deform and vibrate when excited at its resonance frequency. This new regime, working at the fundamental vibration mode, is essentially different from the conventional harmonic/super-harmonic operations. Based on such design, a DFUT could be configured with a nonpiezoelectric elastic membrane with a small groove and a piezoelectric membrane stacked on the top surface, served as the low-frequency CMUT and high-frequency PMUT, respectively. Both the mathematical analysis and COMSOL simulations demonstrate that this configuration could achieve a combination of two independent single-frequency vibration modes. The swept-frequency analysis illustrated that the high-frequency PMUT operation of the proposed DFUT had at least an order-of-magnitude displacement sensitivity improvement compared with the conventional harmonic/super-harmonic operations. In addition, the ratio of two frequencies can be controlled in a wide range of around 2 to more than 20 by changing the dimensions of the groove and piezoelectric membrane. It is noted that this dual-frequency ultrasound operation can be used in any other combination forms, such as pure piezoelectric transducers or pure capacitive transducers. Due to easy controllability of two frequencies, independent optimization for two frequencies, large high-to-low frequency ratio, and easy acoustic impedance matching, the single-element DFUT based device would promote the development of medical imaging and therapy as well as sensing, such as intravascular imaging, photoacoustic imaging, imaging-guided neurosurgery, and HIFU treatments.

## Figures and Tables

**Table 1:** Structural parameters of the single-element DFUT in the glossary of symbols.

Structural Parameter	Symbol
Length of the nonpiezoelectric elastic layer	*l*
Length of the piezoelectric layer	*a*
Thickness of the nonpiezoelectric elastic layer	*t*_1_
Thickness of the middle metal electrode	*t*_2_
Thickness of the piezoelectric layer	*t*_3_
Thickness of the top metal electrodes	*t*_4_
Thickness of the central suspended elastic layer	*t_s_*
Height of the middle plane from the bottom surface	*z_m_*
Distance from the bottom reference surface	*z_i_*

**Table 2:** Specific structural and material parameters of the single-element DFUT.

**Dimensions**	***l* (μm)**	***a* (μm)**	***t*_1_ (μm)**	***t*_2_ (μm)**	***t*_3_ (μm)**	***t*_4_ (μm)**	***t_s_* (μm)**
-	70	20	3	0.15	0.5	0.15	0.5
**Materials**	***E* (GPa)**	***v***	***ρ* (kg/m^3^)**	***ε*_33_**	***S*_11_ (1/Pa)**	***S*_12_ (1/Pa)**	***d*_31_ (pC/N)**
Si	170	0.28	2329	11.7	-	-	-
Pt	168	0.38	21450	2.7	-	-	-
AlN	-	-	-	10.26	2.86 × 10^−12^	−0.90 × 10^−12^	−1.73

**Table 3:** Plate parameter β varying with the length of the square groove for 70 μm DFUT.

Length of the Groove	5 μm	10 μm	15 μm	20 μm	25 μm	30 μm	35 μm	40 μm
β	−0.10510	−0.04133	−0.03703	0.04768	0.23652	0.48654	0.67712	0.78614
